# Harmonizing WHO’s International Classification of Diseases (ICD) and International Classification of Functioning, Disability and Health (ICF): importance and methods to link disease and functioning

**DOI:** 10.1186/1471-2458-13-742

**Published:** 2013-08-12

**Authors:** Reuben Escorpizo, Nenad Kostanjsek, Cille Kennedy, Molly Meri Robinson Nicol, Gerold Stucki, Tevfik Bedirhan Üstün

**Affiliations:** 1 Department of Physical Therapy, Louisiana State University Health Sciences Center, New Orleans, LA, USA; 2 ICF Research Branch in cooperation with the WHO Collaborating Centre for the Family of International Classifications in Germany (DIMDI), Nottwil, Switzerland; 3 Swiss Paraplegic Research (SPF), Nottwil, Switzerland; 4 World Health Organization, Classifications, Terminologies and Standards, Geneva, Switzerland; 5 US Department of Health and Human Services, Office of Health Policy, Washington, DC, USA; 6 Department of Health Sciences and Health Policy, University of Lucerne, Lucerne, Switzerland

**Keywords:** International classification of diseases, ICF, Classification, Functioning, ICD revision, Disability

## Abstract

**Background:**

To understand the full burden of a health condition, we need the information on the disease and the information on how that disease impacts the functioning of an individual. The ongoing revision of the International Classification of Diseases (ICD) provides an opportunity to integrate functioning information through the International Classification of Functioning, Disability and Health (ICF).

**Discussion:**

Part of the ICD revision process includes adding information from the ICF by way of “functioning properties” to capture the impact of the disease on functioning. The ICD content model was developed to provide the structure of information required for each ICD-11 disease entity and one component of this content model is functioning properties. The activities and participation domains from ICF are to be included as the value set for functioning properties in the ICD revision process.

**Summary:**

The joint use of ICD and ICF could create an integrated health information system that would benefit the implementation of a standard language-based electronic health record to better capture and understand disease and functioning in healthcare.

## Background

Describing and understanding the relationship between disease and functioning requires the use of two of the World Health Organization’s classifications systems: the International Classification of Diseases (ICD) [1] and the International Classification of Functioning, Disability and Health (ICF) [2]. The ICD classifies disease entities and other health conditions to gather diagnostic information, while the ICF classifies domains of functioning and disability in terms of body functions and structures or activities and participation at the body, person and societal levels. The ICD and the ICF classification systems are intended by WHO to complement each other so as to capture and provide the full picture of health or health-related state of an individual. Currently, however, there is no standard platform in which the disease and its impact on functioning are concurrently used within an integrated health information system. Efforts to capture the impact of a disease in a structured and systematic way have so far been hampered by the failure to link the ICD and the ICF at a conceptual and operational level.

### ICD revision

The ICD is undergoing its 11^th^ revision (ICD-11) [3] wherein part of the process is to add information from the ICF to the classification of diseases by adding “functioning properties” (i.e. ICF domains or codes) to capture the impact of the disease on functioning. In this integrated system, we want to be able to use universal domains (functioning properties) that depict the functioning of an individual by way of the ICF and also use information related to disease entities (ICD codes).

The process of revising the ICD is coordinated through Topic Advisory Groups (TAGs), each of which is responsible for different content areas. Responsible for the appropriate integration of the functioning properties is the Functioning Topic Advisory Group (fTAG), which consults with each of the TAGs regarding how to deal with functioning properties for their assigned ICD entities.

### Functioning properties of the ICD-11 content model

The ICD-11 Content Model (Table 1) provides the structure of information detail required for each ICD-11 code included through the revision process [3,4]. In the ICF, “functioning” is an encompassing term for body functions, body structures, and activities and participation. In the ICD Content Model at this time, functioning properties only include the activities and participation component of the ICF. *Activity* is defined in the ICF as the “execution of a task or action by an individual”, while *participation* is defined as “involvement in a life situation” [2]. Activities and participation are important in describing the impact of a disease because they capture the broad and relevant aspects of activity and involvement with society and life in general. Table 2 lists the ICF categories that are included in the value set for functioning properties. Hence, an ICD code would have a corresponding value set of functioning properties.

**Table 1:** **The Content Model of the ICD 11 **[4]

**Any category in ICD is represented by: TITLE of ENTITY: Name of disease, disorder, or syndrome**	
**1. ICD Concept Title**	**8. Temporal Properties**
1.1 Fully Specified Name	8.1 Age of Occurrence & Occurrence Frequency
	8.2 Development Course / Stage
**2. Classification Properties**	**9. Severity of Subtypes Properties**
2.1 Parents	
2.2 Type	
2.3 Use and Linearization(s)	
**3. Textual Definition(s)**	**10. Functioning Properties**
	10.1 Impact on Activities and Participation
	10.2 Contextual Factors
	10.3 Body Functions
**4. Terms**	**11. Specific Condition Properties**
4.1 Base Index Terms	11.1 Biological Sex
4.2 Inclusion Terms	11.2 Life-Cycle Properties
4.3 Exclusions	
**5. Body Structure Description**	**12. Treatment Properties**
5.1 Body System(s)	
5.2 Body Part(s) [Anatomical Site(s)]	
5.3 Histopathology	
**6. Manifestation Properties**	**13. Diagnostic Criteria**
6.1 Signs and Symptoms	
6.2 Investigation Findings	
**7. Causal Properties**	**14. External Causes**
7.1 Etiology Type	
7.2 Causal Properties- Agents	
7.3 Causal Properties- Causal Mechanisms	
7.4 Genomic Linkages	
7.5 Risk Factors	

**Table 2:** List of ICF-based functioning properties value set for an ICD code

**Domains**		**ICF codes**
**Understanding**	Watching	d110
	Listening	d115
	Learning	d130-d155
	Focusing attention	d160
	Reading	d166
	Writing	d170
	Calculating	d172
	Solving problems	d175
	Other specified	
**Communication**	Communicating with others	d310
		d315
		d320
		d325
	Speaking	d330
	Starting a conversation	d3500
	Sustaining a conversation	d3501
	Other …	
**Mobility**	Standing	d4104
	Bending	d4105
	Maintaining a body position	d415
	Transferring oneself	d420
	Lifting and carrying objects	d430
	Fine hand use	d440
	Hand and arm use	d445
	Walking short distances	d4500
	Walking long distances	d4501
	Vigorous activities	d455
		d4303
	Moving around within home	d4600
	Moving around outside the home and other buildings	d4602
	Using transportation	d470
	Driving	d475
	Other …	
**Self-Care**	Washing oneself	d510
	Caring for body parts	d520
	Urination	d5300
	Defecation	d5301
	Dressing	d540
	Eating	d550
	Drinking	d560
	Managing one’s health (needs, assistance or oversight)	d570
	Other …	
**Interpersonal Relations**	Making friends	d7200
		d7500
	Engaging with other people	d740
		d750
	Maintaining family relationships	d760
	Dealing with strangers	d730
	Engaging in sexual relationships	d7702
	Other …	
**Life Activities**		
Household	Shopping	d620
	Cooking/preparing meals	d630
	Doing housework	d640
	Looking after/helping others	d660
	Other …	
School	Attending school	d820
	Learning a job (vocational training, apprenticeship)	d825
	Going to university	d830
	Other …	
Work and economic life	Engaging in paid work	d850
	Seeking employment	d8450
	Performing job related tasks	d8451
	Handling money	d860
	Other …	
Life management	Undertaking a single task	d210
	Undertaking multiple tasks	d220
	Carrying out daily routine	d230
	Handling stress and psychological demands	d240
	Other …	
**Social Participation**	Taking part in social life	d910
	Sports	d9201
	Travel	d920
	Visiting friends	d9205
	Human rights (e.g. self-determination, equal opportunities)	d940
	Political life and citizenship (e.g. voting)	d950
	Other …	
**Children and Youth**	Learning to read	d140
	Learning to write	d145
	Learning to calculate	d150
	Communicating with others	d310
		d315
		d320
		d325
	Speaking	d330
	Attending school	d8201
	Taking exams	d8202
	Playing with others	d880
		d9200

### The task of populating the functioning properties in iCAT

Before ICD-11 is completed, functioning properties will need to be populated for each ICD code. This task of population is being done and coordinated using the web-based *International Collaborative Authoring Tool* (iCAT) by content experts worldwide in three steps: [1] selection of functioning properties provided in iCAT (Table 2), [2] if an additional ICF domain or category needs to be added based on a published disease-specific ICF Core Set, then it is entered manually into the iCAT, and [3] use evidence from the literature (i.e. mini-review) by identifying the commonly used measures relevant to the disease of interest, and in those measures identify meaningful concepts of functioning with a focus on activities and participation in life situations, and then subsequently link the identified concepts to a specific domain in the ICF [5].

## Discussion

Obtaining information about disease entities and their impact on functioning is not entirely new in the field of medicine and health. While the consideration of the disease and its impact on functioning has been in place, or at least acknowledged, for a long time, [6] there remain prevailing issues, such as the lack of wide dissemination and implementation extending beyond simple awareness [7,8]. The operationalization of integrated disease-and-functioning models currently varies, is fragmented across healthcare settings, and is perhaps more commonly observed in healthcare systems with medium to advanced infrastructures and access to resources. We can do a better job at facilitating an integrated disease-and-functioning model across systems from low to high resource countries. Moreover, the ongoing ICD revision would make the assessment and documentation of a comprehensive set of information about a disease entity as broad and as inclusive as possible; at the same time utilizing the standard and common language of the ICF on functioning. This information will consist of biomedical and biopsychosocial aspects of the disease that will provide clinicians and users alike an integrated and unified ICD-ICF platform and which will be helpful in interdisciplinary communication towards a concerted planning of care ultimately benefiting the patients.

The ICD-11 is due to be launched in 2015, and steps toward that goal are being pursued. Certainly there are challenges on our way, but there are also opportunities that are presented for users in the clinical and research communities to actively contribute in this huge endeavor by WHO and its collaborators worldwide. The unified ICD-ICF in the ICD-11 will allow for consistent terminologies to be used and to be harmonized across ICD and ICF and will provide holistic information about a disease entity and its impact on the functioning of an individual. Efforts are also currently being taken to facilitate the identification of the overlaps for ICD-11 disease entities and their titles with their conceptual equivalent in the ICF towards harmonization of ICD and ICF.

## Conclusion

The joint use of the ICD and ICF towards an integrated health information model would, in our opinion, benefit medicine and health systems and would support the push for the implementation of a standard language-based electronic health record system towards better health services planning and reimbursement.

## Abbreviations

fTAG: Functioning Topic Advisory Group; iCAT: International Collaborative Authoring Tool; ICD: International Classification of Diseases; ICF: International Classification of Functioning, Disability and Health; TAG: Topic Advisory Group; WHO: World Health Organization.

## Competing interests

The authors declare that they have no competing interests.

## Authors’ contributions

All authors provided concept/idea, consultation, and writing, and reviewed the manuscript before submission. All authors read and approved the final manuscript.

## Authors’ information

RE is Assistant Professor, Department of Physical Therapy, School of Allied Health Professions Louisiana State University Health Sciences Center, New Orleans LA USA; adjunct research scientist at the ICF Research Branch in cooperation with the WHO Collaborating Centre for the Family of International Classifications in Germany (DIMDI), Nottwil, (Switzerland); and the Swiss Paraplegic Research (SPF), Nottwil, Switzerland.

NK is technical officer at World Health Organization, Classifications, Terminologies and Standards (CTS), Department of Health Statistics and Informatics (HSI), Geneva, Switzerland.

CK is with the US Department of Health and Human Services, Office of Health Policy Washington D.C., USA.

MMRN is technical officer at World Health Organization, Classifications, Terminologies and Standards (CTS), Department of Health Statistics and Informatics (HSI), Geneva, Switzerland.

GS is director of the ICF Research Branch in cooperation with the WHO Collaborating Centre for the Family of International Classifications in Germany (DIMDI), Nottwil, (Switzerland) and the Swiss Paraplegic Research (SPF), Nottwil, Switzerland; is Professor and Chair at the Department of Health Sciences and Health Policy, University of Lucerne, Lucerne, Switzerland.

TBU is head of WHO’s Family of International Classifications, Geneva, Switzerland.

R Escorpizo is an employee of the Louisiana State University Health Sciences Center (LSUHSC). This article was developed in his professional capacity and does not necessarily represent the views of LSUHSC.

C Kennedy is an employee of the Office of the Assistant Secretary for Planning and Evaluation (ASPE), Department of Health and Human Services (HHS). This article was developed in her professional capacity and does not necessarily represent the views of ASPE or HHS.

## Pre-publication history

The pre-publication history for this paper can be accessed here:

http://www.biomedcentral.com/1471-2458/13/742/prepub
